# Trends in the incidence and survival of patients with esophageal cancer: A SEER database analysis

**DOI:** 10.1111/1759-7714.13311

**Published:** 2020-03-10

**Authors:** Haiqi He, Nanzheng Chen, Yue Hou, Zhe Wang, Yong Zhang, Guangjian Zhang, Junke Fu

**Affiliations:** ^1^ Department of Thoracic Surgery The First Affiliated Hospital of Xi'an Jiaotong University Xi'an China

**Keywords:** esophageal cancer, incidence, prevalence, SEER, survival

## Abstract

**Background:**

Recent studies have indicated that the incidence of esophageal cancer has declined in the past decade in the U.S. However, trends in the incidence and survival have not been thoroughly examined.

**Methods:**

Data from 46 063 patients with esophageal cancer between 1973 and 2015 were collected from the Surveillance, Epidemiology, and End Results database. The trends in the age‐adjusted incidence and survival were analyzed using joinpoint regression models.

**Results:**

The age‐adjusted incidence of esophageal cancer increased from 5.55 to 7.44 per 100 000 person‐years between 1973 and 2004. Later, it decreased at an annual percentage change of 1.23%. In the last 40 years, the strong male predominance increased slightly. Importantly, the percentage of patients with localized stage of squamous cell cancer decreased. It was observed that the incidence of esophageal squamous cell carcinoma declined since 1986, while the incidence of esophageal adenocarcinoma sharply increased since 1973 and surpassed the rate of squamous cell cancer, mainly due to the increase in the incidence among men. Consistently, the estimated 40‐year limited‐duration prevalence of esophageal adenocarcinoma was higher than that of esophageal squamous cell carcinoma. Additionally, we observed a modest but significant improvement in survival during the study period.

**Conclusion:**

The incidence of esophageal squamous cell carcinoma has decreased significantly over the past four decades in the U.S., while the incidence of adenocarcinoma has increased, particularly among men. Overall, the long‐term survival of patients with esophageal cancer is poor but it has improved over the past decades, especially for the localized disease.

**Key points:**

**Significant findings of the study**

The incidence of esophageal cancer has decreased at an annual percentage change of 1.23% since 2004. The incidence of esophageal adenocarcinoma has sharply increased since 1973 and surpassed the rate of squamous cell cancer, mainly due to the increase in the incidence among men.

**What this study adds**

There has been a shift in the prevalence of esophageal cancer histological subtypes over the past decades in the U.S. We found that the incidence of esophageal squamous cell carcinoma has continued to decrease, while the esophageal adenocarcinoma rate has continued to increase.

## Introduction

Esophageal cancer is the eighth most common malignancy in the world with an estimated 455 800 new cases (3.2% of the total cancer cases) in 2012, and the sixth leading cause of cancer‐related death with an estimated 400 200 deaths (4.9% of the total deaths).^1^ Worldwide incidence rates vary by more than 21‐fold among different regions, with the highest rates seen in eastern Asia and eastern and southern Africa and the lowest rate in western Africa.^1^ The main histological types of esophageal cancer are squamous cell carcinoma and adenocarcinoma, with esophageal squamous cell carcinoma (ESCC) being the predominant histological type worldwide.^2^ However, the distribution of the histological types of esophageal cancer has been changing in the last few decades. The incidence of esophageal adenocarcinoma (EAC) has been increasing in the last five decades, becoming the most common esophageal malignancy in Western countries.^3^, ^4^, ^5^

In the U.S., the age‐adjusted rate of EAC increased steadily from 1975 to 2000s, while the rate of ESCC declined.^6^, ^7^ However, there are few reports evaluating whether this trend has persisted. Therefore, the current study examined the temporal trends in the incidence and long‐term survival of esophageal cancer from 1973 to 2015 using the Surveillance, Epidemiology, and End Results (SEER) program database.

## Methods

### Data source

The data of patients with esophageal cancer were obtained from the SEER database. The SEER database is the only comprehensive source of population‐based information in the U.S., which is maintained by the National Cancer Institute. In this study, we obtained the incidence and survival data of patients with esophageal cancer from the original registry sites (Connecticut, Iowa, New Mexico, Utah and Hawaii, and the metropolitan areas of Atlanta, Detroit, San Francisco‐Oakland and Seattle‐Puget Sound). Specifically, these nine SEER registries have the longest follow‐up duration information and include data since 1973.

All cases of esophageal cancer were selected from the SEER database using tumor site codes ranging from C15.0 to C15.9. Our analysis was limited to patients who were at least 20 years of age at the time of diagnosis. Specifically, in this study, histological subtypes were classified as adenocarcinoma (8140–8141, 8143–8145, 8190–8231, 8260–8263, 8310, 8401, 8480–8490, 8550–8551, 8570–8574, 8576), squamous cell carcinoma (8050–8078, 8083–8084), and others, according to the International Classification of Disease for Oncology (ICD‐O) version 3.^8^ Tumor stage was evaluated by the SEER historic staging system (localized, regional, distant and unknown), allowing for consistent cancer staging across all study years.

### Data analysis

Incidence rates were calculated per 100 000 person‐years, and age‐adjusted to the 2000 U.S. standard population. Age‐adjusted incidence rates with 95% confidence intervals (CIs) were analyzed by sex, histology, race, age groupings (20–44, 45–49, 50–54, 55–59, 60–64, 65–69, 70–74, 75–79, 80–84 and 85+ years), and SEER stage. The male‐to‐female ratio among the esophageal cancer patients was calculated from the patient count. For survival analysis, only the cases with microscopically confirmed primary esophageal cancer were included, and the cases which had unknown age, were identified by autopsy or a death certificate or cases without follow‐up records were excluded. The incidence and relative survival rates were calculated using SEER*Stat software (version 8.3.4). The joinpoint regression model^9^ was used to calculate the annual percentage change (APC) in esophageal cancer incidence and survival. The joinpoint regression analysis uses a statistical algorithm to identify the best‐fitting points where the statistically significant changes occur. We used the Joinpoint Regression Program, Version 4.5.0.1 (distributed by the Statistical Applications and Research Branch of the National Cancer Institute) to calculate APC in the incidence or five‐year survival rates. Up to three joinpoints were allowed in the models. For the five‐year overall survival trend analysis of EAC, we selected survival data from 1975 to 2015 due to zero values in 1974. The Kaplan‐Meier method with a log‐rank test was used to compare the overall survival of patients between the groups.

Limited duration prevalence analysis was achieved by the counting method using SEER*Stat software. Only cases with first malignant primaries and known age were selected, and cases with the only death certificate and autopsy were excluded. We project SEER limited‐duration prevalence to the U.S. population by matching race, sex, and age, and sum them together using ProjPrev (Version 1.0.4. June 2017; Data Modeling Branch, National Cancer Institute). The January 1, 2015, US populations were estimated by averaging 2014 and 2015 populations.

## Results

### Overall incidence trends and prevalence

**Figure 1:**
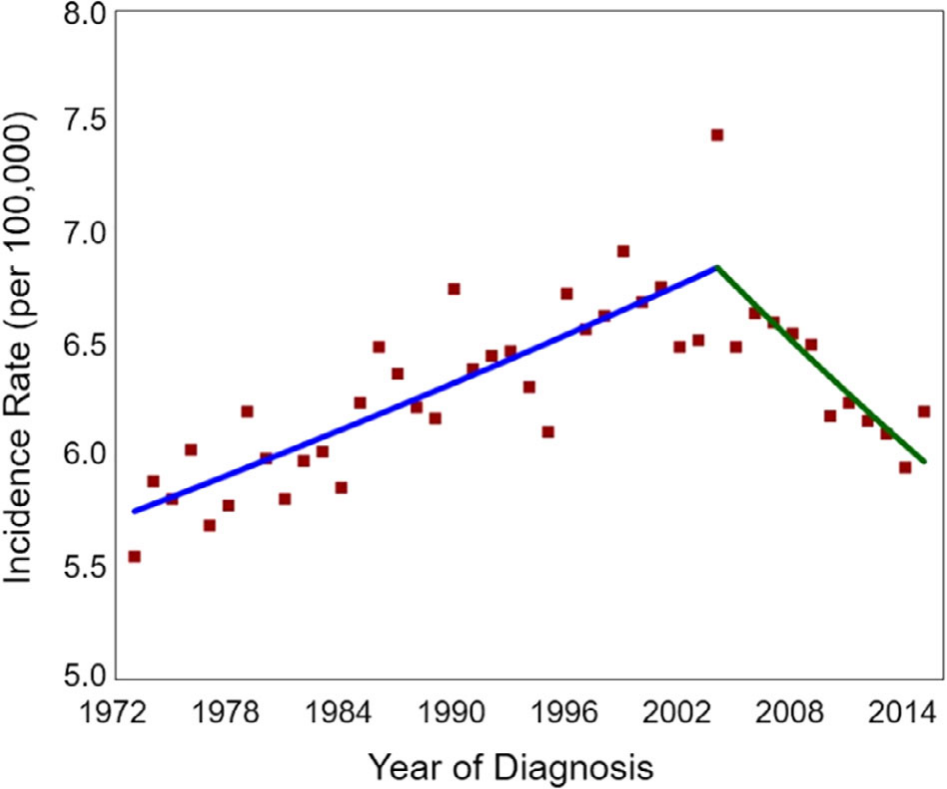
Joinpoint analysis of the incidence rates of esophageal cancer in the U.S. between 1973 and 2015. (^) Indicates the annual percent change (APC) that is significantly different from zero (*P* < 0.05). 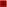 observed, 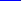
1973–2004 APC = 0.56^, 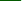 2004–2015 APC = −1.23^.

A total of 46 063 cases met the specified criteria and were included in the incidence analysis. Male (34 275 [74%]) and white patients (36 593 [79%]) comprised the majority of the study population. The overall age‐adjusted incidence rate of esophageal cancer was 6.33 per 100 000 person‐years. Specifically, it increased from 5.55 to 7.44 per 100 000 person‐years between 1973 and 2004 with an APC of 0.56% (*P* < 0.001). Later, the incidence of esophageal cancer has declined at a statistically significant APC of 1.23%. In 2015, the age‐adjusted incidence rate of esophageal cancer was 6.20 per 100 000 person‐years (Fig 1).

Among the population sampled by the SEER 9 registries, the estimated 40‐year limited‐duration prevalence for esophageal cancer on 1 January 2015, was 3461 (95% CI, 3347–3578). The estimated 40‐year limited‐duration prevalence for ESCC was 911 (95% CI, 853–972), that for EAC was 2306 (95% CI, 2213–2402). We projected this data to the US standard population and matched by race, sex, and age. The estimated 40‐year limited‐duration prevalence for esophageal cancer on 1 January 2015, in the United States, was 39 616.

### Incidence stratified by sex

**Figure 2:**
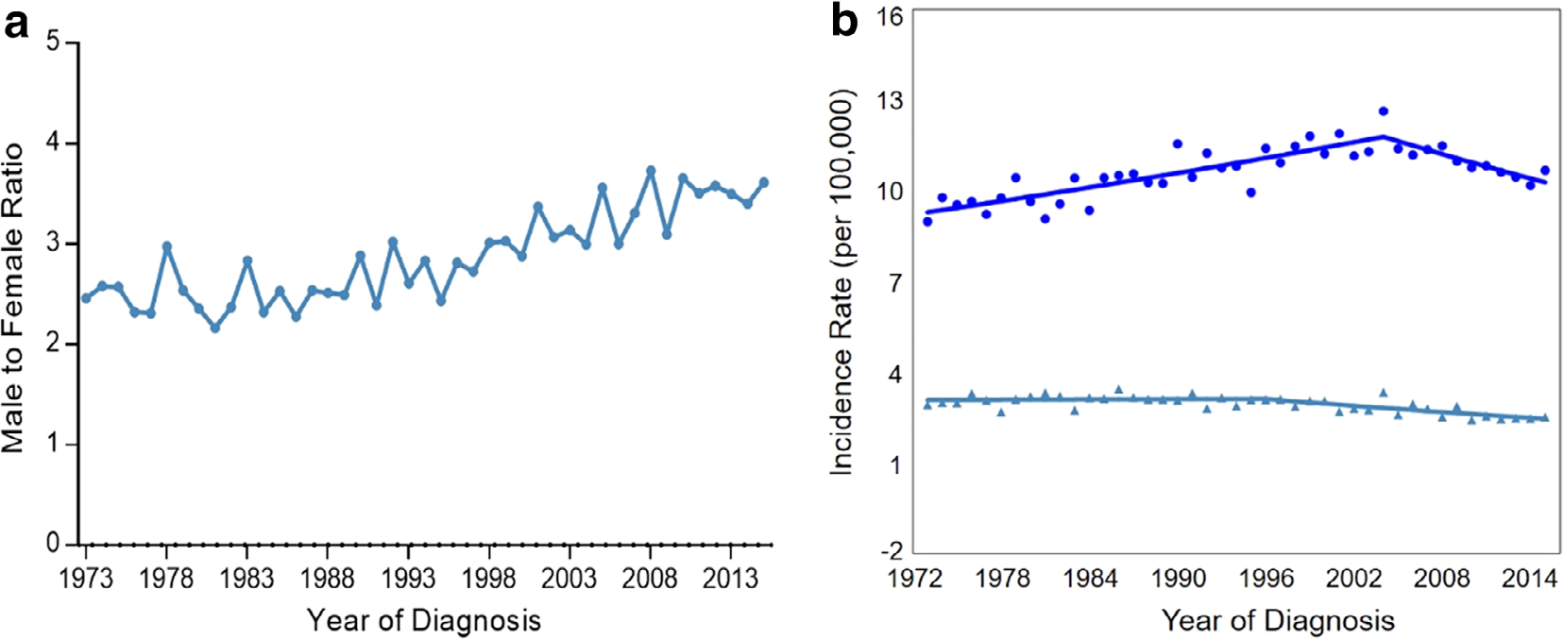
Incidence trends of esophageal cancer stratified by sex in the U.S. (1973–2015). (**a**) The male‐to‐female ratio of patients with esophageal cancer was calculated from the absolute count of male and female patients. (**b**) Joinpoint analysis of the incidence rates of esophageal cancer stratified by sex. 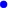 Male – 1 Joinpoint, 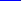 1973–2004 APC = 0.77^, 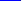
2004–2015 APC = −1.24^. 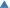 Female –_Joinpoint, 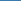
1973–1996 APC = −0.04^, 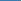
1996–2015 APC = −1.24^. (^) Indicates the annual percent change (APC) that is significantly different from zero (*P* < 0.05).

Data analysis indicated that esophageal cancer was more commonly diagnosed in men than in women, at the ratio of 2.91:1. This strong male predominance among the esophageal cancer patients increased slightly in the past 40 years (men‐to‐women ratio was 2.46:1 in 1973 and 3.62:1 in 2015), although the data fluctuated annually (Fig 2a). We also analyzed the incidence of esophageal cancer stratified by sex. Joinpoint analysis showed that the incidence of esophageal cancer in men increased at an APC of 0.79% between 1973 and 2004. In contrast, the rate of esophageal cancer in women remained stable between 1973 and 1996. Afterwards, the rate of esophageal cancer in both men and women decreased at an APC of 1.24% (Fig 2b).

### Incidence stratified by age and stage

**Figure 3:**
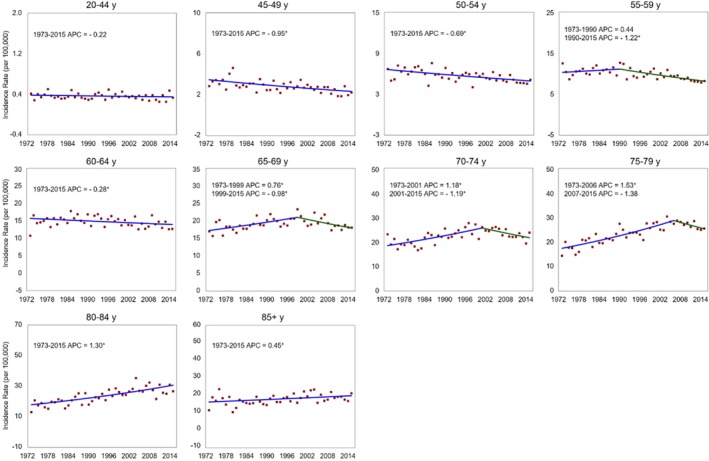
Annual percent change (APC) in age‐specific esophageal cancer incidence rates in the U.S. (1973–2015). An asterisk indicates the APC that is significantly different from zero (*P* < 0.05).

When the incidence rates were examined across age groups, we discovered that the esophageal cancer incidence rates were declining (between 1973 and 2015) in patients younger than 65. Conversely, the rates were increasing in patients older than 65 years from 1973 through the early 2000s. Later, a declining trend was observed in adult age groups at 65–69 years (APC = 0.98%), 70–74 years (APC = 1.19%), and 75–79 years (APC = 1.38%). Interestingly, the rates were increasing in patients who were 80 years or older in the study period (Fig 3).

**Figure 4:**
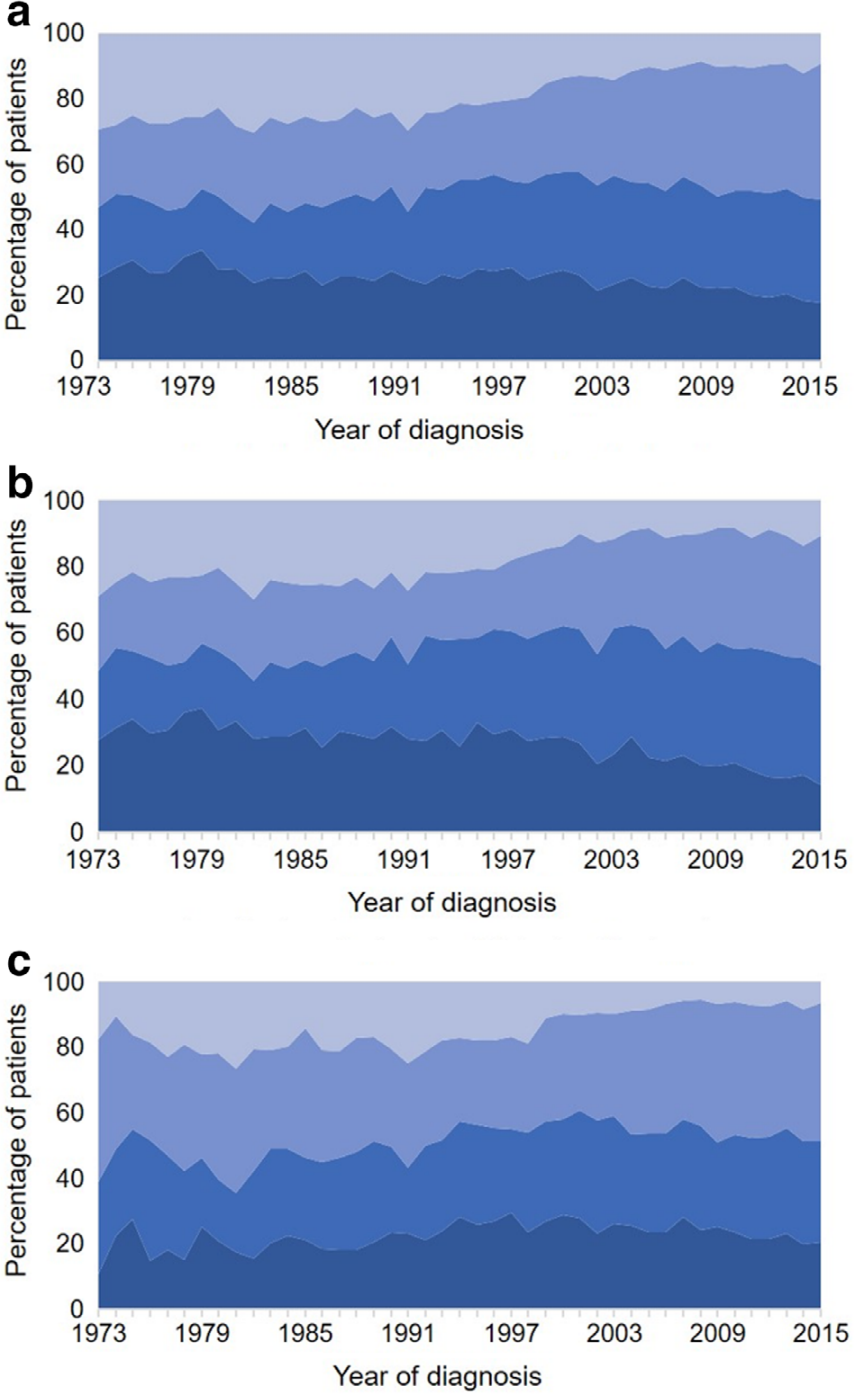
The SEER stage distribution of esophageal cancer cases diagnosed in the U.S. (1973–2015). The percentage of patients represented by localized, regional, distant, and unstaged subtypes were calculated for patients with (**a**) esophageal cancer, 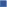 Localized, 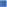 regional, 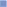 distant, 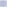
unstaged. (**b**) esophageal squamous cell carcinoma, 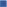 Localized, 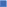 regional, 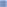 distant, 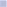
unstaged. and (**c**) esophageal adenocarcinoma. 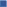 Localized, 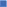 regional, 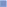 distant, 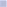
unstaged.

During the study period, the percentage of esophageal cancer patients with staged disease increased, whereas the percentage of unstaged cancer patients decreased from 29.09% to 9.17% (Fig 4a). Surprisingly, the percentage of ESCC patients with localized disease stage decreased, while it slightly increased in patients with EAC (Fig 4b,c).

### ESCC and EAC

**Figure 5:**
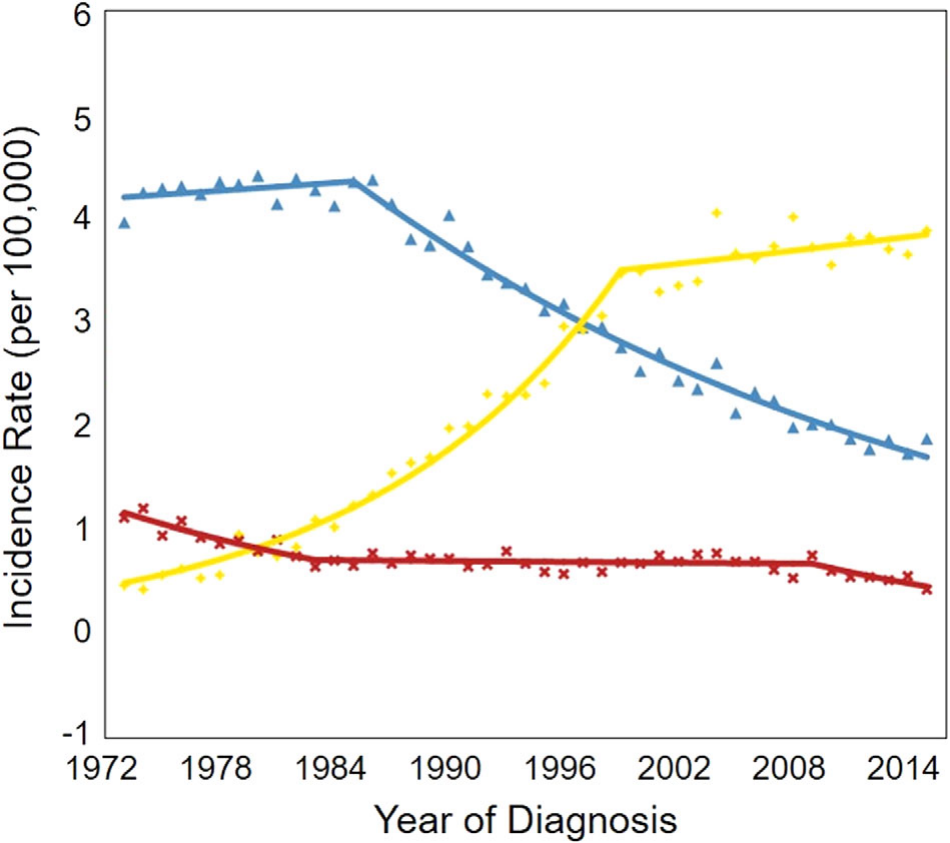
Joinpoint analysis of the incidence rates of esophageal cancer stratified by the histological type in the U.S. (1973–2015). (^) Indicates the annual percent change (APC) that is significantly different from zero (*P* < 0.05). 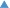
Squamous – 1 Joinpoint, 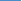 1973–1985 APC = 0.30, 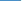 1985–2015 APC = −3.08^; 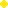
Adenocarcinoma – 1 Joinpoint, 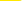
1973–1999 APC = 7.82^, 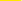
1999–2015 APC = 0.58^; 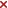 other – 2 joinpoints, 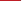 1973–1983 APC = −4.80^, 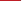 1983–2009 APC = −0.19, 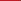 2009–2015 APC = −6.30^.

**Figure 6:**
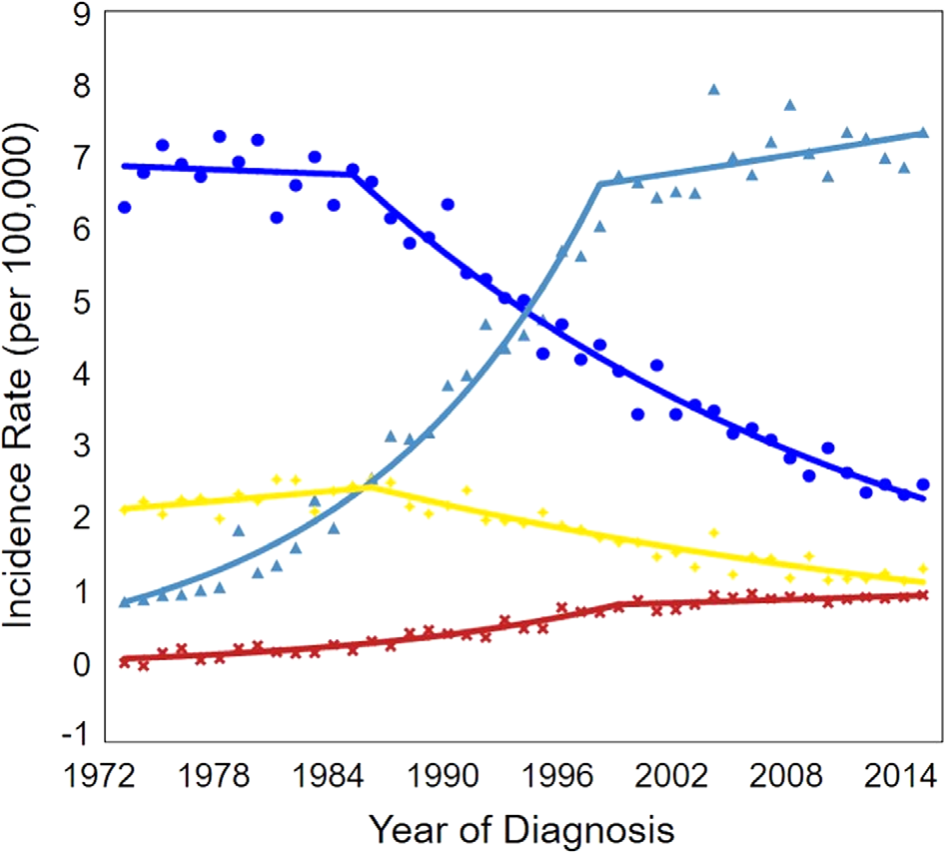
Joinpoint analysis of the incidence rates of esophageal cancer stratified by sex and histological type in the U.S. (1973–2015). (^) Indicates the annual percent change (APC) that is significantly different from zero (*P* < 0.05). 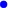
Male/Squamous – 1 Joinpoint, 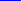
1973–1985 APC = −0.15, 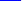
1985–2015 APC = −3.49^; 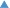
Male/Adenocarcinoma – 1 Joinpoint, 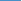
1973–1998 APC = 8.25^, 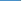
1998–2015 APC = 0.59^, 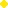
Female/Squamous – 1 Joinpoint, 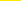
1973–1986 APC = −0.96, 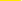
1986–2015 APC = −2.52^, 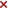
Female/Adenocarcinoma – 1 Joinpoint, 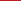
1973–1999 APC = 7.27^, 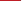
1999–2015 APC = 0.84.

We analyzed the incidence of esophageal cancer stratified by histological subtypes. Joinpoint analysis revealed that the incidence of ESCC remained stable between 1973 and 1986, and was higher compared to patients with EAC. After 1986, the incidence of ESCC steadily declined at an APC of 3.08% (Fig 5). In contrast, the incidence of EAC increased sharply at an APC of 7.82% between 1973 and 1999. This occurred mostly due to the increase in the incidence among men (Fig 6), eventually surpassing the rate of ESCC. After 1999, the rate increased modestly at a statistically significant APC of 0.58% (Fig 5). However, the rate of EAC among women was still lower compared to the rate of ESCC, even though it also increased during the past 40 years (Fig 6).

### Survival trends

**Figure 7:**
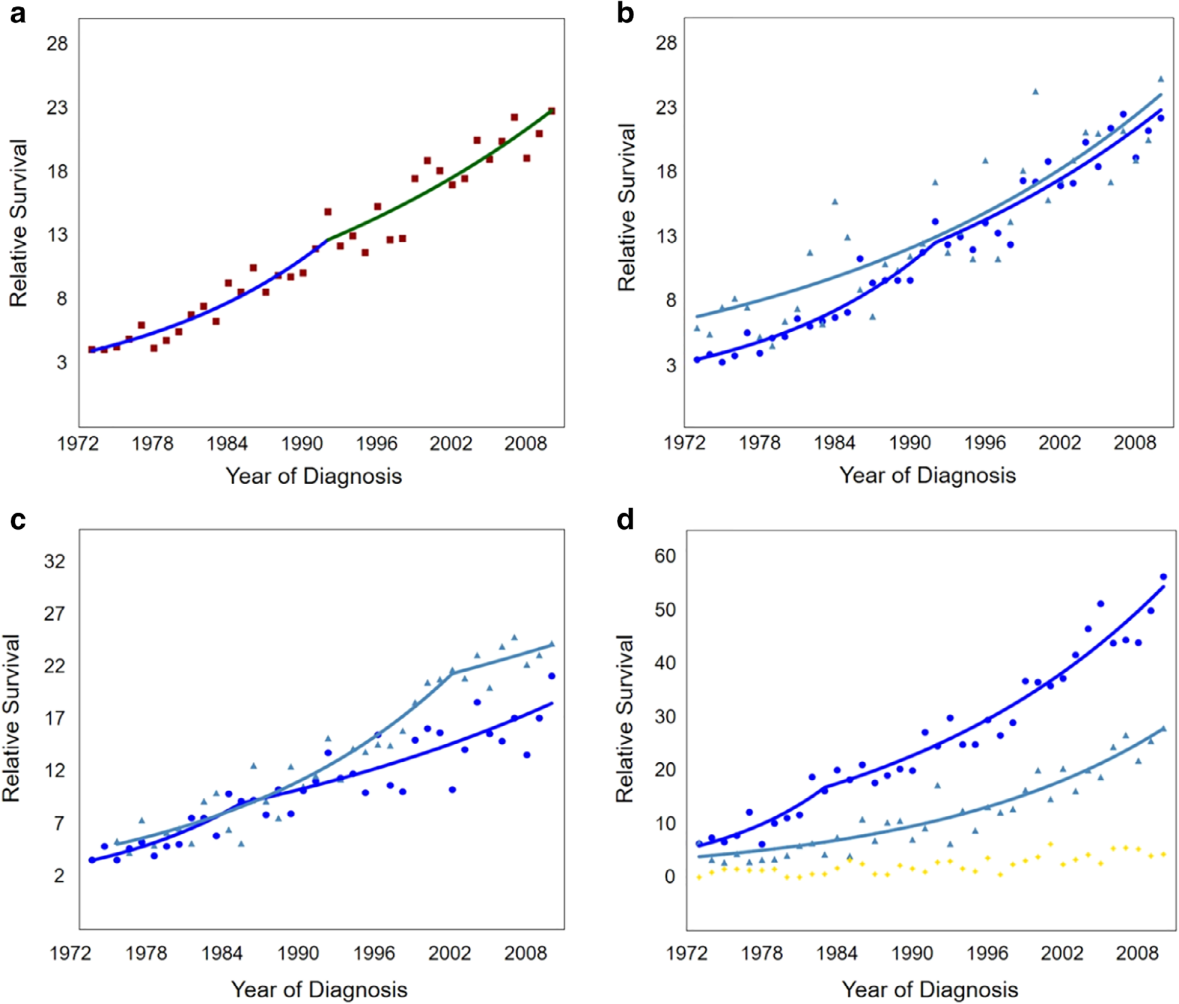
Joinpoint regression analysis of the five‐year survival trends in (**a**) esophageal cancer, 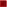 observed, 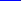 1973–1992 APC = 6.28^, 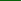
1992–2010 APC = 3.31^, and stratified by (**b**) sex, 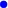 Male – 1 Joinpoint, 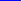
1973–1992 APC = 7.19^; 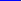
1992–2010 APC = 3.42^, 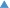
Female – 0 Joinpoints, 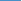
1973–2010 APC = 3.52^, (**c**) histology, 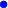
Squamous – 1 joinpoint, 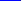
1973–1985 APC = 8.02^, 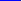
1985–2010 APC = 2.92^, 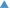
Adenocarcinoma – 1 Joinpoint, 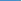
1975–2002 APC = 5.44^, 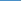
2002–2010 APC = 1.50, or (**d**) stage, 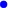
Localised – 1 Joinpoint, 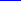
1973–1983 APC = 11.06^, 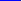
1983–2010 APC = 4.45^, 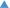
Regional – 0 Joinpoints, 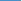
1973–2010 APC = 5.48^, 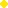
Distant – invalid data. (^) Indicates the annual percent change (APC) that is significantly different from zero (*P* < 0.05).

We analyzed the five‐year all‐cause survival trends in patients with esophageal cancer. The five‐year survival rate was 14.3% in all patients. Joinpoint analyses indicated that the five‐year survival rate showed an increasing trend throughout the monitoring period, with an APC of 6.28% from 1973 to 1992 and an APC of 3.31% from 1992 to 2010 (Fig 7a). When stratified by sex, the five‐year survival rates were higher in women (Fig 7b), mainly due to the higher survival of female patients with squamous cell carcinoma (Figure S1).

The five‐year survival rate for patients with ESCC increased from 3.6% to 21.1% between 1973 and 2010. The five‐year survival rate for patients with EAC increased from 5.4% to 24.2% across the same time period (Fig 7c). There was no significant difference in the five‐year survival rates between patients with ESCC and EAC between 1973 and 1990s (APC = 8.02% for ESCC, APC = 5.44% for ESCC) (Fig 7c). After 1985, the increasing trend slowed down for ESCC patients (APC = 2.92%) and the survival rates for EAC patients became higher compared to ESCC patients. Importantly, the APC of survival for EAC patients was not statistically significant between 2002 and 2010.

Survival is closely related to esophageal cancer staging. Joinpoint analysis indicated a significant increase in the five‐year survival rate for both localized and regional stages between 1973 and 2010 (APC = 11.06% and 4.45% for the localized disease before 1983 and after 1983, respectively; APC = 5.48 for the regional stage) (Fig 7d). However, the five‐year survival trends for patients with distant stage cancer could not be calculated due to zero values in the five‐year survival rates for some years.

## Discussion

ESCC and EAC appear to be distinct from each other in epidemiology, biology, and prognosis. Previous studies indicate the increasing rates of EAC and decreasing rates of ESCC in the U.S.^10^ Here, we provide an update on the recent incidence and survival changes between 1973 and 2015 using the population‐based SEER database. Our study suggested that there has been a shift in the prevalence of esophageal cancer histological subtypes over the past decades in the U.S. We found that the incidence of ESCC continued to decrease, while the EAC rate continued to increase. Consistently, the prevalence of EAC was higher than that of ESCC.

The incidence of esophageal cancer varies by region, with the highest rates occurring in Asia.^11^ Although ESCC is the dominant type worldwide, the incidence rates of EAC in the Western world have been increasing in the last halfcentury, making EAC more prevalent than ESCC.^3^, ^4^, ^5^ Similarly, the prevalence of EAC is also increasing in some Asian countries such as Singapore.^12^ This temporal trend is predicted to continue in the upcoming few decades.^13^ Interestingly, we observed that the incidence rate of EAC began to slow down during the recent years in the U.S., although it is yet to reach a plateau.

Previous studies observed a decrease in the incidence of esophageal cancer in the non‐White population within the U.S.^7^ Using recent data from the SEER database, we updated the incidence trends of esophageal cancer and demonstrated that the overall incidence has been declining since 2004, which is mainly due to the continuing decline in the ESCC incidence. Important risk factors for ESCC include tobacco use, excessive use of alcohol, low socioeconomic status, and low intake of fruits and vegetables.^14^, ^15^ Interestingly, a previous study suggested that tobacco smoking and alcohol consumption together account for 58% of ESCC cases overall, with 78% and 38% of ESCC cases occurring in men and women, respectively.^16^ We suspect that the declining incidence of ESCC in the U.S., as observed in our study, may be partly due to the reduced consumption of alcohol and the lower prevalence of smoking.^17^, ^18^ The higher prevalence of these two factors in men can also partly explain the sex disparity in ESCC rates.

Our results indicate that EAC surpassed ESCC and became the most prevalent type of esophageal cancer in the U.S. This burden of EAC underscores the need for raising EAC awareness and promoting interventions that will reduce the risk of developing the disease. The known risk factors for EAC include obesity, gastroesophageal reflux disease (GERD), Barrett's esophagus (BE), tobacco use, and a diet low in vegetables and fruits.^14^ Among these factors, obesity is likely the most important risk factor for EAC.^19^ The sharp increase of the EAC incidence in the U.S. can be partly explained by the increasing numbers of overweight or obese populations.^20^, ^21^ Another important risk factor for EAC is GERD, which is associated with obesity.^22^, ^23^ As a sequela of GERD, BE is the only known precursor lesion for EAC. Specifically, in BE, the stratified squamous epithelium of the esophagus is replaced by the metaplastic columnar epithelium. When compared with the general population, BE increases the risk of EAC by approximately 30‐fold.^24^ A previous study showed that there was a linear increase in the incidence of diagnosed BE in the general population in Western countries.^25^ This increasing prevalence of BE is likely to result in the increasing rates of EAC in the next few decades. Consequently, the strategy for the prevention and control of EAC relies on upper endoscopy coupled with biopsy to identify BE in patients with GERD and regular endoscopic surveillance in BE patients.^26^

Our study showed that the strong male predominance has increased slightly over the past decades in the U.S. The incidence trend in women is difficult to interpret in the context of changes in exposure to known risk factors. To date, the factors driving the influence of sex in esophageal cancer incidence have not been identified. A previous study demonstrated that the influence of sex hormones was a likely candidate explaining the sex difference in esophageal cancer incidence.^27^

The prognosis among patients diagnosed with esophageal cancer is poor. The present study shows that the overall five‐year survival rates of esophageal cancer have increased in the examined study period. We believe that this is not only due to the improvement in surgical techniques and adjuvant therapy, but also due to the early diagnosis of an increased number of esophageal cancer cases owing to the increasing use of upper gastrointestinal endoscopy.^26^, ^28^ Interestingly, the five‐year survival rates for EAC were higher compared to ESCC, especially after 1992, which is consistent with the previous studies.^29^, ^30^ This may be due to the decrease in the percentage of patients with localized squamous cell carcinoma, as observed in our analysis. In theory, regular endoscopic surveillance in BE patients may increase the proportion of localized EAC, but our analysis indicated that the proportion of localized EAC did not significantly change during the study period. Future studies should address the potential causes of changing trends in survival in both histological types of esophageal cancer.

In summary, esophageal cancer is one of the most common cancers in the world with a poor prognosis. Using more recent data from the SEER database, we found that the incidence of EAC continues to rise and it has become the main histological type of esophageal cancer in the U.S. Although the increase in the incidence of EAC has slowed down, it has not yet reached a plateau. Importantly, the survival rate of patients with esophageal cancer, especially that with EAC, has greatly improved in the past few decades.

## Disclosure

The authors report no conflicts of interest in this work.

## Supporting information


**Appendix S1.** Supporting information
