# Supplementary material for: Trends in the incidence and survival of patients with esophageal cancer: A SEER database analysis
Source: Thorac Cancer. 2020 Mar 10;11(5):1121–8. doi: 10.1111/1759-7714.13311 (PMC7180574; doi:10.1111/1759-7714.13311)
Supplement: Supplementary file 1 — Appendix S1. Supporting information [file TCA-11-1121-s001.docx]

**Trends in the incidence and** **survival of patients with esophageal cancer: a SEER database analysis**

Haiqi He^1^, MD; Nanzheng Chen^1^, MD; Yue Hou^2^, MD; Zhe Wang^1^, MD; Yong Zhang^1^, MD; Guangjian Zhang^1^, MD; and Junke Fu^1^, MD


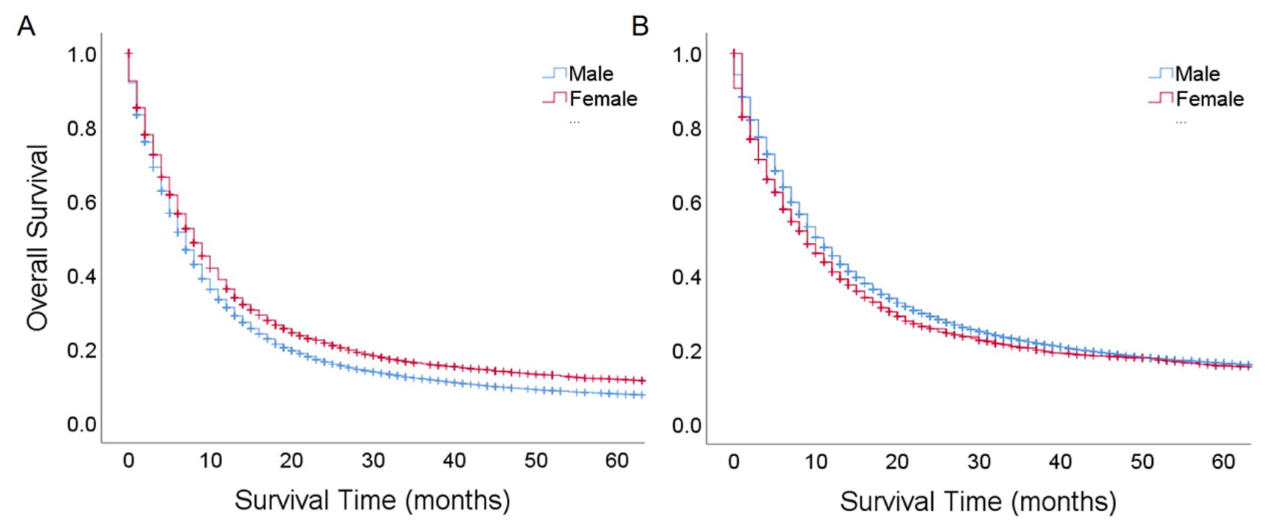


**Figure S1**. Kaplan–Meier survival curves for (A) esophageal squamous cell cancer and (B) esophageal adenocarcinoma. The curves are stratified by sex.
